# Artificial Intelligence-Driven Neuromodulation in Neurodegenerative Disease: Precision in Chaos, Learning in Loss

**DOI:** 10.3390/biomedicines13092118

**Published:** 2025-08-30

**Authors:** Andrea Calderone, Desirèe Latella, Elvira La Fauci, Roberta Puleo, Arturo Sergi, Mariachiara De Francesco, Maria Mauro, Angela Foti, Leda Salemi, Rocco Salvatore Calabrò

**Affiliations:** 1 IRCCS Centro Neurolesi Bonino-Pulejo, S.S. 113 Via Palermo, C.da Casazza, 98124 Messina, Italy; desiree.latella@irccsme.it (D.L.); roccos.calabro@irccsme.it (R.S.C.); 2 Department of Clinical and Experimental Medicine, University of Messina, Piazza Pugliatti, 1, 98122 Messina, Italy; elvira.lafauci@gmail.com (E.L.F.); roberta.puleo@hotmail.com (R.P.); arturosergi@gmail.com (A.S.); marydefra26@gmail.com (M.D.F.); mariamauro722@gmail.com (M.M.); angela-foti@alice.it (A.F.); leda.salemi96@gmail.com (L.S.)

**Keywords:** artificial intelligence, neuromodulation, neurodegenerative diseases, Alzheimer’s disease, Parkinson’s disease, multiple sclerosis, deep brain stimulation, transcranial magnetic stimulation, transcranial direct current stimulation, vagus nerve stimulation

## Abstract

Neurodegenerative disorders such as Alzheimer’s disease (AD), Parkinson’s disease (PD), and multiple sclerosis (MS) are marked by progressive network dysfunction that challenges conventional, protocol-based neurorehabilitation. In parallel, neuromodulation, encompassing deep brain stimulation (DBS), transcranial magnetic stimulation (TMS), transcranial direct current stimulation (tDCS), vagus nerve stimulation (VNS), and artificial intelligence (AI), has matured rapidly, offering complementary levers to tailor therapy in real time. This narrative review synthesizes current evidence at the intersection of AI and neuromodulation in neurorehabilitation, focusing on how data-driven models can personalize stimulation and improve functional outcomes. We conducted a targeted literature synthesis of peer-reviewed studies identified via PubMed, Embase, Scopus, and reference chaining, prioritizing recent clinical and translational reports on adaptive/closed-loop systems, predictive modeling, and biomarker-guided protocols. Across indications, convergent findings show that AI can optimize device programming, enable state-dependent stimulation, and support clinician decision-making through multimodal biomarkers derived from neural, kinematic, and behavioral signals. Key barriers include data quality and interoperability, model interpretability and safety, and ethical and regulatory oversight. Here we argue that AI-enhanced neuromodulation reframes neurorehabilitation from static dosing to adaptive, patient-specific care. Advancing this paradigm will require rigorous external validation, standardized reporting of control policies and artifacts, clinician-in-the-loop governance, and privacy-preserving analytics.

## 1. Introduction

Neurodegenerative disorders are diseases in which the cells of the central nervous system stop working or die [1]. These disorders, including Alzheimer’s disease (AD), Parkinson’s disease (PD), and multiple sclerosis (MS), share several morphological and pathophysiological features and, critically, a common pattern of irreversible neuronal degeneration [2,3]. Neurodegenerative diseases are characterized as chronic, progressive, and incurable conditions and may result in tremendous morbidity, poor quality of life, and notable mortality rates [4]. The neuropathogenesis of neurodegenerative diseases is thought to involve various factors, including accumulation of protein aggregates, microglial-mediated neuroinflammation processes, and dysregulation of neurotransmitter systems, leading to an insidiously progressive loss of cognitive, motor, and/or autonomic function [5,6,7]. Neurological diseases affected approximately 3.4 billion people in 2021; neurodegenerative disorders constitute a substantial subset of this overall burden and are among the costliest to society, according to The Lancet Neurology and World Health Organization data [8,9]. In the same year, incident cases were estimated at 6.19 million for dementia, 0.57 million for PD, 1.54 million for MS, and 0.04 million for idiopathic epilepsy [9]. The incidence continues to rise globally, especially in low- and middle-income countries, with women consistently showing higher age-standardized rates for both dementia and PD [9,10]. In this difficult epidemiological context, neurorehabilitation is a crucial mainstay of care for patients with neurodegenerative diseases. Yet, conventional rehabilitative strategies frequently fail to address the complex, dynamic aspects of these needs, necessitating the development of novel interventions that promote neuroplasticity, restore functional abilities, and ultimately improve quality of life. Of the novel approaches being developed, neuromodulation is prioritized for its possible direct effects on neural activities and patterns of recovery [11]. Neuromodulation techniques refer to a family of medical technologies that aim at modifying nervous system functioning by precisely directing an electrical, chemical, or mechanical stimulation to specific neural targets [12]. The main goal is to re-establish some physiologic equilibrium in the neural networks destabilized by pathological processes or trauma [13]. Neuromodulation encompasses both invasive and non-invasive modalities; among the most studied in brain rehabilitation are vagus nerve stimulation (VNS), typically invasive in its implanted form, with transcutaneous variants, and transcranial direct current stimulation (tDCS), which is non-invasive [12,13,14,15]. They work modulating neuronal firing probability, shaping synaptic plasticity, and promoting the reorganization of neural networks to facilitate functional recovery following different neurological diseases [16,17]. Although neuromodulation shows strong potential as a treatment modality, optimal stimulation profiles of intervention parameters, patient stratification, and specific mechanisms underlying clinical responses are areas of active research. Concurrently, artificial intelligence (AI) has evolved quickly as transformative in medicine and neuroscience. It represents the development and application of computational algorithms, often inspired by the architecture and function of biological neural networks, that enable machines to perform tasks traditionally requiring human intelligence [18]. AI comprises a vast range of modalities in the neurorehabilitation field that includes machine learning—(ML), deep learning—(DL), and neural network-based systems that can detect complex patterns in data, predict outcomes, and aid in clinical decision-making [19]. AI models can process neuroimaging, electrophysiological, and clinical data with a level of speed and accuracy that is unprecedented, and in doing so, diagnose neurodegenerative conditions earlier and with more reliability [20,21].

Bringing these trajectories together, the convergence of AI and neuromodulation offers a timely and conceptually powerful response to longstanding challenges in neurorehabilitation [22]. Continuous sensing (neural, kinematic, and behavioral) can feed AI models that infer latent disease states and recommend or enact adaptive control policies; in turn, neuromodulatory devices can close the loop by adjusting dose, timing, or spatial targeting on the fly while preserving clinician oversight. Such systems promise several gains: (i) better patient selection via biomarker-guided stratification, (ii) individualized parameter tuning that reduces side effects and improves efficacy, (iii) real-world monitoring that anchors therapy to daily function rather than episodic snapshots, and (iv) learning health system workflows that improve with use, generating evidence at the point of care. Nonetheless, realizing this vision requires addressing interpretability, safety, privacy, and equity from the outset and embedding multidisciplinary governance into design and deployment. These opportunities and constraints motivate the present narrative review. Our purpose is threefold. First, we synthesize recent evidence at the intersection of AI and neuromodulation in neurorehabilitation for AD, PD, and MS, mapping the landscape from foundational methods to translational exemplars. Second, we critically appraise clinical readiness: where closed-loop systems, decision-support tools, or AI-optimized programming have demonstrated feasibility or benefit; where limitations in data quality, external validity, or usability remain; and what implementation considerations (artifact handling, power/computation constraints, staffing, reimbursement) will shape uptake. Third, we identify cross-cutting gaps and outline research priorities that can accelerate safe, equitable, and effective integration into routine practice. We also highlight open datasets and toolchains to enable reproducible benchmarking and clinician–engineer collaboration In doing so, we aim to move beyond technology-centric descriptions toward a clinically grounded framework that connects algorithms and devices to patient-centered outcomes.

To meet these aims, we proceed from broad principles to specific applications. We first review neuromodulation modalities and their clinical role, highlighting the strengths and limitations that motivate AI-enabled augmentation. We then survey AI methodologies with an emphasis on those capable of handling longitudinal, multimodal data and supporting adaptive control. Next, we examine applied use cases across diagnosis, monitoring, outcome prediction, and closed-loop therapy, drawing out disease-specific themes and shared architectural elements. Finally, we discuss translational, ethical, and health system implications with a forward-looking agenda that prioritizes rigorous validation, transparent and interpretable controllers, standardized reporting, and cost-effectiveness, alongside patient safety and equity. Finally, we aim for this review to function as a practical bridge across disciplines. For clinicians, it distills actionable considerations for patient selection, monitoring cadence, and parameter adjustment that can inform everyday programming and follow-up. For AI specialists, it frames clinically salient problem formulations, data characteristics, and reporting standards to prioritize translational relevance over benchmark performance alone. For device engineers, it highlights constraints that matter at the bedside, as well as artifact suppression, power budgets, secure telemetry, and explainable controllers. For trialists and regulators, it outlines candidate endpoints, transparency requirements, and evidence templates suited to adaptive systems. Finally, for health system leaders, it maps implementation and reimbursement inflection points. In short, we provide a shared vocabulary and reference architecture to accelerate safe, equitable adoption.

## 2. Neuromodulation in Neurodegenerative Disease: Foundations and Clinical Innovations

Neuromodulation has emerged as a core therapeutic paradigm of contemporary neurorehabilitation, delivering targeted interventions that modulate neural activity and foster recovery across a broad spectrum of neurological disorders. These approaches apply external electrical, magnetic, or chemical stimulation to recalibrate circuit excitability and plasticity, thereby restoring network integrity and improving clinical outcomes. Principal modalities include deep brain stimulation (DBS), transcranial magnetic stimulation (TMS), tDCS, and VNS. Each modality differs in its mechanism of action, clinical indications, degree of invasiveness, strength of supporting evidence, and practical considerations for implementation [23].

### 2.1. Deep Brain Stimulation: Circuit-Based Therapies for Complex Neurological Symptoms

Deep brain stimulation (DBS) is a complex invasive surgical procedure that has revolutionized the treatment of some movement disorders and is now under investigation for other, wider neurologic and psychiatric diseases [24]. DBS requires the stereotactic implantation of electrodes (leads) into defined deep brain structures, most often the subthalamic nucleus, globus pallidus internus, or ventral intermediate nucleus of the thalamus [25,26,27]. Pulse generators are linked with electrodes, the electrodes implanted in the target areas of the brain, that will provide continuous and programmable electrical stimulation through an implantable pulse generator, generally subcutaneously placed in the thoracic cavity [28]. PD is the leading clinical indication for DBS, and it is considered the most effective surgical therapy for medication-resistant motor manifestations, including tremor, rigidity, bradykinesia, and severe motor response fluctuations [29]. Strong results of randomized controlled trials (RCTs) and long-term cohort studies have convincingly shown substantial gains in motor function, quality of life, and medication use for appropriately selected patients [30,31,32,33,34]. DBS is also a therapy approved by the Food and Drug Administration (FDA) for essential tremor, dystonia, and obsessive–compulsive disorder, with continued investigation for effectiveness in patients with epilepsy, Tourette’s syndrome, and major depressive disorder [35,36,37]. The mechanism of action of DBS is complex and incompletely understood. It is not as simple as producing a lesion but is increasingly recognized to have immediate and long-term effects on neural networks that include not only the suppression of pathological oscillations but also changes in neurochemistry and the facilitation of synaptic plasticity [38]. Neuroprotective and even neurogenic effects are now detected in some preclinical models. The clinical realization of adaptive DBS, adapting stimulation parameters according to real-time changes in physiology, likely frames the biggest leap in personalized neuromodulation to date, both with regard to efficacy and reduction in adverse events [39]. However, DBS is not without its challenges. It is an invasive procedure with known surgical complications, including infection, hemorrhage, and hardware-related problems [40,41]. The safe and effective delivery of DBS requires prudent patient selection, multidisciplinary perioperative care, and careful long-term device programming. DBS is still not available to many because of cost and the necessity of available complex centers and close follow-up. Its potential in managing non-motor and neuropsychiatric symptoms is an area of promise, requiring better-powered studies.

### 2.2. Transcranial Magnetic Stimulation: Expanding Clinical Horizons in Neurorehabilitation

Transcranial magnetic stimulation (TMS) is a non-invasive neuromodulation technique that modulates cortical excitability by delivering focally targeted magnetic fields [42]. TMS has been studied predominantly in relation to neurodegenerative diseases, specifically PD and AD, and less commonly in MS. In PD, repetitive TMS (rTMS) of the motor cortex and supplementary motor area has been shown to improve motor symptoms, including bradykinesia, rigidity, and gait disorders [43]. There are several RCTs and meta-analyses that confirm the adjuvant beneficial role that rTMS plays in ameliorating motor deficits, and the greatest effect is with high-frequency stimulation in general [44,45,46]. Additionally, TMS may have a positive impact on non-motor symptoms in PD, depression, and cognitive impairment, though with modest and variable effects [47,48]. For AD and mild cognitive impairment (MCI), chronic high-frequency rTMS to the dorsolateral prefrontal cortex has demonstrated promise for enhancing cognitive performance, including memory-related, executive, and language domains [49,50]. Studies combining rTMS and cognitive training demonstrate the additive nature of the two treatments, but it is not known to what extent this benefit endures or what the optimal stimulation regimes are [51]. The application of TMS in MS is less established, but there is some early evidence for improving fatigue and some cognitive deficits [52,53]. Both single and repeated courses of TMS have been reported to enhance neuroplasticity, facilitating the reorganization of neural circuits and modulating neurotransmitter systems implicated in neurodegeneration [54,55]. Access issues, costs of treatment, and the requirement for multiple sessions present pragmatic barriers to implementation in this population [56]. From a mechanistic standpoint, contemporary models converge on rTMS engaging N-Methyl-D-Aspartate (NMDA)-dependent long-term potentiation/long-term depression-like plasticity with downstream modulation of GABAergic/glutamatergic balance and neurotrophin signaling, alongside network-level changes measurable with TMS-electroencephalograms (EEG) and magnetic resonance imaging (MRI) connectivity [57,58,59]. In AD-relevant networks, perturbational markers (TMS-EEG evoked potentials/oscillations) track enhanced frontoparietal effective connectivity after prefrontal high frequency (HF)-rTMS and may serve as treatment biomarkers [60]. In PD, a meta-analysis indicates that stimulating the supplementary motor area (SMA) yields significant motor improvement versus sham, and an RCT shows that HF-rTMS was more effective than SMA in alleviating freezing of gait [61,62]. Safety is favorable under guideline-concordant parameters; the International Federation of Clinical Neurophysiology update reports a very low seizure risk, and pooled estimates suggest 8 per 100,000 sessions overall, <2 per 100,000 within recommended dosing; common adverse effects are transient scalp discomfort and headache [63,64]. Health–economic analyses, mostly in depression, show that rTMS can be cost-effective or cost-saving versus medication over 12 months, but analogous evaluations in PD/AD are scarce [65]. Real-world session costs range from hospital production estimates of around EUR 125–EUR 130 per session in France to higher private tariffs in the UK; capital costs for clinical systems typically span roughly USD 50,000–USD 200,000, creating access and reimbursement barriers for neurodegenerative indications [66].

### 2.3. Transcranial Direct Current Stimulation in Practice: Pathways to Clinical Impact

Transcranial direct current stimulation (tDCS) is a noninvasive neuromodulatory technique where a low-level direct current is delivered to the scalp to moderately alter the excitability of neurons, promoting neuroplasticity [67]. Although evidence continues to accumulate for the use of tDCS for AD and PD, in the case of MS, evidence is only recently emerging from the few published studies that exist [68]. With regard to AD, one of the more exciting recent advances is that anodal tDCS applied over prefrontal or temporoparietal cortical regions is not only safe in the short term but may enhance multiple cognitive domains, including memory, attention, and global cognitive scores [69,70,71,72]. And when tDCS is combined with cognitive training, additional processing suggests that it can offer small but genuine aid to those with MCI [73]. But these cognitive gains typically do not persist for long and can even disappear after the stimulation stops [74,75]. The same applies for PD, where anodal-tDCS has been tested in the motor but also prefrontal area [76,77]. Optimal montages and dosing remain to be established. Small studies suggest reductions in fatigue and improvements in attention and working memory in people with MS [78,79,80]. To summarize, tDCS is an extremely interesting treatment. It is very safe, comfortable, portable, and cost-effective, making it well suited for long-term or home use, especially for chronic neurodegenerative diseases [81]. Mechanistically, tDCS produces subthreshold shifts in neuronal membrane potential and modulates synaptic efficacy via NMDA-dependent plasticity while altering intracortical excitation–inhibition; accumulating preclinical and translational work also implicates astrocytic/microglial responses (gliotransmission, Ca^2+^-dependent signaling, cytokine modulation) as potential mediators of processes relevant to neurodegeneration [82,83,84,85]. AD studies report small improvements in global cognition and language enhancement when paired with cognitive training, but durability beyond 1–3 months is inconsistent [86]. In PD, the up-to-date narrative and quantitative literature suggests overall small or limited effects, with a focused meta-analysis showing modest gains in gait/balance, particularly when combined with rehabilitation [87,88]. In MS, remotely supervised/home-based programs are advancing; a randomized, sham-controlled trial pairing home tDCS with cognitive training reduced fatigue with good adherence and safety, building on earlier remote-supervision paradigms [89,90]. Adverse effects are generally mild, including tingling, itching, erythema, and transient headache/fatigue, with rare skin burns (improper preparation) and occasional mood elevation/mania reported in vulnerable individuals; serious neurologic events are exceedingly uncommon [91,92]. From an implementation and cost perspective, tDCS devices are low-cost and portable (research-grade/home-use typically costs hundreds to low thousands of USD), enabling tele-supervised at-home delivery that minimizes travel and chair time; preliminary economic signals from tele-tDCS programs suggest feasibility at low program costs (e.g., USD 750 in a U.S. home-administered series), though formal cost-utility analyses in PD/AD/MS are still limited [93,94].

### 2.4. Vagus Nerve Stimulation: Bridging Central and Peripheral Modulation

Vagus nerve stimulation (VNS), delivered via implanted or external devices to modulate central-peripheral circuits [95], is established for refractory epilepsy and depression, but its role in neurodegeneration remains exploratory [96]. In PD and AD, VNS has been tested mainly as an adjunct, with preliminary signals for improved motor performance (including tremor) and possibly non-motor symptoms, though adequately powered randomized trials are still needed [97,98,99]. Mechanistically, VNS may act via neurotransmitter release, dampening neuroinflammation, and promoting plasticity [100]; in AD, putative benefits relate to enhanced noradrenergic and cholinergic transmission with potential cognitive effects, yet available studies show only small, inconsistent gains in attention and memory [101,102]. Evidence in MS is nascent and largely preclinical, with no human efficacy data to date [103,104]. Given the surgical risks for implants, there is a clear need to validate non-invasive protocols and uncertain long-term benefits [105]. Despite mechanistic plausibility in noradrenergic/cholinergic and anti-inflammatory pathways [106], VNS should presently be considered investigational in neurodegenerative disorders.

### 2.5. Limitations and Challenges of Conventional Neuromodulation

However, neuromodulation also has significant limitations, despite its considerable enhancement of the neurorehabilitation field. There is limited understanding of the underlying mechanisms in all modalities, which impacts the development of personalized protocols and ultimately patient selection. It frequently needs to be empirically programmed by trial and error rather than designed through a rational process [107,108]. In neurodegenerative populations specifically, the current evidence base is marked by small-to-moderate effect sizes, short follow-up, heterogeneous protocols, frequent reliance on surrogate outcomes, underpowered RCTs, and limited generalizability beyond highly selected cohorts, making the clinical relevance of gains uncertain. The effectiveness these strategies may have, except for the pursuit of a few of the indications, remains relatively uncertain, and the clinical relevance of the improvements is, especially for tDCS and VNS, dubious. The invasiveness, cost, and need for expertise restrict the accessibility of some of these therapies, notably DBS or implanted VNS, for treatment in low- and middle-income areas. Additionally, practice-level barriers include fragmented reimbursement pathways, capital and maintenance costs, workforce training and credentialing, scheduling burden (multi-session courses), travel/transport limitations, and adherence challenges in patients with cognitive/psychiatric comorbidities; for home or tele-supervised use, caregiver support, digital literacy, and data governance further constrain implementation. The absence of a standardized protocol, notably for tDCS, hinders both research synthesis and clinical transfer [67]. Key methodological gaps include insufficient head-to-head comparative-effectiveness studies, unclear dose–response and maintenance/booster schedules, and limited use of active comparators integrated with best-practice rehabilitation, which collectively impede translation. Adverse effect profiles are well tolerated and are modality- and patient population-dependent. Nevertheless, systematic safety surveillance and device registries are needed to refine risk estimates across indications and delivery settings, including device upkeep for implanted systems and skin/comfort monitoring for non-invasive approaches. Another issue is how to integrate neuromodulation into the current rehabilitation strategy. These modalities are rarely administered as stand-alone treatments and achieve their maximum effectiveness when included as components of such patient-centered programs of therapeutic exercise for the entire spectrum of functional loss [109]. Accordingly, pragmatic implementation studies that embed neuromodulation within multidisciplinary pathways (physiotherapy/occupational therapy/cognitive rehabilitation) and define referral, dosing, and tapering algorithms are a pressing priority. Furthermore, the development of adaptive, closed-loop, and biomarker-based neuromodulation, typically involving AI and state-of-the-art signal processing, is a clear frontier, with the potential to offer increased precision, efficacy, and safety. Future research should prioritize biomarker-guided stratification (e.g., TMS-EEG, peripheral inflammatory markers), state-dependent/closed-loop control, target selection and montage optimization, multicenter pragmatic trials with durable patient-centered outcomes (≥6–12 months), and robust health–economic evaluations to inform equitable reimbursement and access, including in low- and middle-income settings. Table 1 provides some additional key clinical and practical aspects of the neuromodulation techniques discussed in this paragraph [110,111].

## 3. The Role of Artificial Intelligence in Neuroscience and Rehabilitation

AI is one of the most transformative influences on the present state of neuroscience and neurorehabilitation, particularly within the context of neurodegenerative conditions such as AD, PD, and MS. With an increasing incidence of these disorders in aging populations, there is a greater necessity for more accurate diagnosis, monitoring, and treatment [112]. AI is a multipurpose, powerful instrument because of its unprecedented accuracy in analyzing large, heterogeneous databases, which generates new understanding of disease processes and pipelines for care optimization. It also enables a hyper-drive of patient-centered ‘personalized’ care and tailoring and de-risking of business models in healthcare systems with new evidential bases for decisions, outcomes, and values [113]. AI is a research tool that is evolving very quickly to become a strong part of clinical practice in neurorehabilitation, with great potential effects on patients’ outcomes. In practical terms, this shift is visible in three domains that cut across diseases: (i) large-scale, real-world remote assessments that bring objective measures of mobility and cognition into routine care [114]; (ii) low-burden digital biomarkers such as speech-derived features that can flag prodromal cognitive change [115,116]; and (iii) learning health system workflows that continuously refine risk stratification and follow-up using multimodal data streams [117].

### 3.1. State-of-the-Art AI Methodologies in Neurorehabilitation

The core of the AI revolution in neuroscience is a number of interlocking approaches. At the root of these fields is ML, which is a set of algorithms that can learn from data and make predictions/decisions without being explicitly programmed [118]. One of ML’s subfields, DL, uses deep (multi-layered) neural networks modeled after the brain’s hierarchical mechanism of information processing [119]. One specific DL, a type of convolutional neural networks (CNN), greatly improves the ability to analyze imaging data segmentation, classification, and feature extraction from MRI and positron emission tomography (PET) scans [120]. Such models have increasingly played a role in identifying the presymptomatic phase of neurodegeneration and in monitoring the rate of brain atrophy [121,122]. Recurrent neural networks (RNNs), and, in particular, long short-term memory (LSTM) networks, are well-suited to model sequence data and are thus well-suited for longitudinal biosignals like EEGs or streams of data collected from wearable sensors [123]. Hybrid architectures using both CNN and LSTM components can explore both spatial and temporal structures of the complex clinical data, like gait analysis for PD or cognitive trajectory prediction in AD [124,125]. In addition to these, ensemble learning methods, including random forest classifiers, have shown good performance in clinical prediction, such as risk models in MS [126,127]. Natural language processing (NLP) is an emerging approach to extract information from the unstructured electronic health record text to facilitate large-scale retrospective research and real-time clinical support [128]. These kinds of generative AI models, with the ability to generate realistic neural or clinical data, are now enabling hypothesis testing, simulating rare diseases, and creating novel possibilities for clinical trial design [129]. Make-or-break advancements in neuromorphic computing, where hardware is designed to mimic the spiking behavior of brain cells, are poised to lower the energy costs of AI-driven neurotech while also speeding up the instantaneous computations that are critical for closed-loop neurorehab systems [130]. Underpinning these methodologies is not just a technological advance but a philosophical one: a move from hypothesis-driven, reductionist inference to data-driven, holistic exploration [131]. Insofar as they reflect the complexity of the brain, the organ they seek to simulate, AI technologies are particularly well adapted to the intrinsic diversity and dynamic progression of neurodegenerative disease. Recent community benchmarks underscore translational readiness; a global ML challenge on wearable signals delivered high-specificity algorithms for freezing-of-gait detection aligned with expert-labeled ground truth, illustrating how CNN/LSTM ensembles can be stress-tested before deployment [132]. Similarly, multimodal pipelines that fuse MRI with clinical/wearable features are now predicting DBS-related outcomes and explaining inter-patient variability, paving the way for objective, biomarker-informed programming without repeating disease-specific results covered later [133].

### 3.2. Applications of AI in Diagnosis, Patient Monitoring, and Outcome Prediction

The application of AI is also increasingly being incorporated through the entire rehabilitation progressive care continuum for neurodegenerative disorders, rewriting established conventions and allowing functionalities not practicable with automated analytics. Among the first successful and influential applications of AI is neuroimaging-based diagnosis. For example, advanced ML models trained on large MRI datasets in AD have demonstrated performance comparable to expert radiologists, and in some retrospective analyses, non-inferior or modestly superior, in detecting early preclinical atrophy and in predicting progression from MCI to dementia in held-out test sets [134]; however, prospective head-to-head evaluations and external validation remain limited. Such models take imaging, genetic, and clinical data into account to provide individualized, nuanced risk predictions [135]. In PD, ML/DL models applied to the analysis of structural/functional imaging and of digital biomarkers (e.g., patterns of movement or voice) have shown the ability to differentiate PD from atypical parkinsonian syndromes, predict disease progression, and personalize the choice of candidates for advanced therapies such as DBS [136,137,138]. AI also has a pivotal role in MS, which includes the automation of lesion detection and quantification in volume on MRI, yielding reliable and reproducible tools for diagnosis, staging, and assessment of treatment response [139,140]. In each of these scenarios, AI’s own ability to synthesize multimodal information (radiographic, fluid biomarker, genomic, and clinical) allows the generation of composite risk scores that more accurately represent disease burden and trajectory than any single value [141,142]. The potential of AI to go further than diagnosis, to continuous, real-time surveillance, is a transformational leap for conditions involving insidious, variable decline. Wearable technologies such as accelerometers, gyroscopes, and biosensors continuously generate large volumes of patient data for cloud-based AI platforms [143]. For PD, ML models can be trained on movement and gait data to identify early instability or dyskinesia, enabling rapid changes in therapy and fall reduction [144,145]. With MS, AI continuously monitors mobility and cognitive function, catching subtle changes that might initially slip past the human eye in episodic clinic visits [146]. In the case of comorbid epilepsy (more prevalent in advanced AD and MS), seizure prediction studies that use AI-driven EEG analytics can detect preictal patterns to predict seizures and reinforce the perspective of prompt intervention and patient safety [147]. This type of home-based AI-powered monitoring is therefore transforming the care of chronic neurodegenerative diseases towards more proactive and responsive care. AI also aids with remote assessment via patient-reported outcomes, speech, and facial expression, all applicable in telemedicine and real-world applications [148]. The most disruptive AI application is treatment outcome forecasting and personalized therapy. ML and DL models integrate a variety of patient data, demographic, genetic, neuroimaging, and sensor output, to predict disease progression, risk of comorbidities, and response to treatments [149,150]. The same models can predict functional recovery for stroke survivors with pre-existing neurodegenerative pathology to help adapt individualized rehab protocols [151,152]. In MS, AI can predict the risk of relapse and can predict the anticipated response to treatments among disease-modifying therapies, which can be used for shared decision-making [153]. There are now AI-enabled robotic exoskeletons, as well as virtual reality (VR)-based neurorehabilitation systems that modify the intensity of therapy and the content of therapy in real-time depending on how the patient is performing, in an attempt to optimize both engagement and neuroplasticity [154,155]. To illustrate current clinical traction without duplicating later sections, three concrete examples are noteworthy. Firstly, smartphone-based “Floodlight MS” tasks have been validated for remote measurement of hand motor function, gait/postural stability, and cognition, supporting frequent, low-burden monitoring in naturalistic settings [114,156]. Secondly, speech-based models can augment conventional AD work-ups by detecting subtle lexical-prosodic changes associated with early cognitive decline [115,116]. Thirdly, community-validated wearable algorithms for freezing-of-gait provide standardized, reproducible endpoints for PD motor instability, facilitating therapy titration and trial design [132]. At the same time, rigorous synthesis reminds us that translation requires standardized image analysis and external validation; reviews of automated MS lesion/atrophy tools emphasize workflow integration, scanner effects, and reporting standards as preconditions for routine clinical use [157].

### 3.3. Advantages and Limitations of AI Compared to Traditional Analytics in Neurorehabilitation

The application of AI in neurorehabilitation is a hallmark of an encompassing new line of approach and constitutes a notable alternative to classical analytical methods when the fit with the brain robustness and flexible pattern recognition characteristics is concerned; however, it also marks a series of challenges to be taken into account. Firstly, there is the ability of AI to offer a dispassionate analysis, which might be the headline benefit. Because AI models are trained directly on large, diverse sets of data, they can help reduce the subjective variability and interpretive bias that can be inherent in human judgment [158]. Particularly in a neurodegenerative disease, there is value to be gained from such an objective approach because subtle initial signs might not otherwise be noticed by a clinician, or they might not be seen similarly by all observers [159]. As for another strength, AI is a scalable technology. The capacity to analyze and integrate large quantities of high-dimensional data (such as advanced neuroimaging and genomics and continuous sensor streams) goes well beyond what is humanly feasible. This scalability is the basis for achieving precision medicine in neurorehabilitation, in tailoring of interventions to the unique clinical profiles and disease course of individuals, all the more relevant in diseases with large heterogeneity and unpredictability [160]. On top of that, just the way personalization can be so game-changing. Unlike classic protocol-driven solutions, AI-based rehabilitation solutions are able to adjust consistently to a patient’s changing functional state, potentially optimizing therapy on the fly through individual response patterns [161]. This flexibility offers hope for the clinician in preventing, in an efficient and clinically sound way, the patient from losing of the benefits of the treatment over time. In addition, AI-powered tools are enabling patients to receive expert care in a more widespread and cost-effective manner, across remote monitoring solutions and automated triaging tools; providing expert-level supervision to communities with suboptimal or no access to skilled neurorehabilitation professionals has the potential to begin to narrow the gap in availability for high-quality neurorehabilitation services for decades of disparity [162]. Practically, AI-based solutions facilitate automation in clinical workflows, where a lot of time-consuming and repetitive tasks such as image analysis, documentation, and patient monitoring must be managed. It also reduces administrative burden and allows more patient-centered care (by reducing the administrative burdens that inhibit patient-focused care, AI permits clinicians to spend their time where it is most valuable) [163]. Another clear advantage is the predictability of AI. Sophisticated models are able to detect subtle patterns and risk factors for clinical decline of the kind that leads to falls, hospitalizations, or loss of cognitive function that are generally outside the noticing range of humans [156]. It represents a move from reactive to proactive investment intervention that may change the healthcare course of many individuals with neurodegeneration through preventive intervention. But, for all of these revolutionary results, there are also a number of constraints that are restricting the applications of AI [164]. These systems stand and fall with good quality, diverse, and representative data. When collected and curated inadequately, small or bias-ridden datasets can lead to flawed outputs or even cascading imbalances rather than solutions [165]. Furthermore, the interpretability of most state-of-the-art AI models, or the so-called “black box” problem, is still a challenge to be tackled. The lack of transparency of these models may pose limitations for clinical trust, regulatory trust, and the meaningful integration of AI recommendations into daily clinical practice [166]. There are also ethical challenges, such as concerns regarding the privacy and security of sensitive health data and the potential for algorithmic bias. Among at-risk individuals, like those with cognitive impairments, this issue is even more critical, as these individuals may have a diminished capacity to consent to AI-augmented procedures or comprehend their implications [167]. Lastly, we cannot assume that these AI technologies will be readily applied in current clinical practice. Adoption is contingent not just on the strong and transparent validation of new tools but also on thoughtful user interface design, clinician training, and continued support [168]. The use of AI within a neurorehabilitation team provides an invaluable opportunity to augment and enhance the quality of individual neurorehabilitation but should be considered as a support for more nuanced clinical reasoning rather than a surrogate for the clinical expert. Reflecting these realities, recent syntheses urge prospective, multi-site impact studies, explicit bias audits, and reporting standards for external validation and distribution shift so that quantitative MRI and digital endpoints can deliver reproducible value in day-to-day neurorehabilitation without preempting clinician judgment [117,157]. Collectively, these strengths and limitations set the stage for Section 4, which examines how AI-integrated, closed-loop neuromodulation enables real-time, adaptive, and patient-specific interventions across PD, AD, and MS.

## 4. A New Clinical Paradigm: AI-Integrated, Adaptive Neuromodulation in PD, AD, and MS

In recent years, the intersection of ML and AI with neuromodulation has been a driving force behind a new paradigm in clinical management of neurodegenerative disorders such as PD, AD, and MS. Although neuromodulatory interventions, including DBS, tDCS, VNS and rTMS, have broadened therapeutic options in these conditions, their clinical effectiveness has historically been limited by the static nature of the stimulation paradigms and the large inter-individual variability in clinical response. To address this gap, AI-enabled systems increasingly link continuous sensing (neural, kinematic, or behavioral) to adaptive control policies that modulate dose and timing on the fly, prioritizing energy efficiency and symptom-state specificity while preserving clinician oversight [169,170]. Such a timely integration of AI metrics and advanced ML, DL, and multimodal datamining tools, now affords rapid adaptation in the personalization of neuromodulation that is serving to underpin a basic game-changer in real-time, adaptive neuromodulation of both motor and cognitive performance. In this section, we deliver a critical summary of the state-of-the-art developments of AI-based neuromodulation, with a focus on recent progress and translational potentials in PD, AD, and MS. Throughout this section, we emphasize concrete clinical exemplars, implementation constraints (e.g., artifact management, battery/compute limits), and the specific AI methods underpinning each approach, including supervised learning, deep neural networks, and emerging reinforcement learning for closed-loop control [171,172].

### 4.1. Adaptive and Closed-Loop Neuromodulation Systems

The transition from open-loop to adaptive and closed-loop neuromodulation systems is a milestone in the development of individualized treatments for neurodegenerative diseases. In PD, the most prominent application of this paradigm shift is adaptive DBS (aDBS), in which stimulation is modulated on the basis of online neural or kinematic biomarkers. Clinical devices such as the Medtronic Activa (Manufacturer: Medtronic; City/Country: Minneapolis, MN, USA) PC+S^®^ and Percept™ PC, and investigational devices such as the Nexus-D platform, can now record and stimulate local field potentials simultaneously, facilitating closed-loop modulation that can adapt to the state of PD symptoms [173]. Preclinical and clinical studies indicate robust gains in motor symptom management and that closed-loop systems are capable of greater reductions in the severity of tremor but with less power consumption than open-loop DBS [174,175,176,177]. Recent multicenter and chronic investigations, including the ADAPT-PD (aDBS in PD) program have operationalized biomarker-based policies that adjust stimulation from subthalamic beta-band power, demonstrating feasibility, energy savings, and signals of clinical benefit over conventional DBS [169,175,176]. A simple real-time policy many readers can visualize uses a dual-threshold “hysteresis” rule; if beta power rises above an upper limit for a brief window, amplitude increases and if it falls below a lower limit, amplitude decreases, buffering rapid oscillations and minimizing unnecessary stimulation [175,178]. Importantly, translating AI-enabled tDCS from the clinic to the home requires addressing feasibility, safety, and regulatory/ethical guardrails in tandem. Feasibility hinges on remote workflows that include user training, caregiver support where needed, automated pre-session checks (e.g., impedance thresholds, montage verification by app prompts), and tele-supervision for troubleshooting and adherence auditing. Safety must be engineered into the stack: hard limits on dose per session/day, lockout periods, skin-integrity prompts with photo/video spot-checks, automatic shut-off on abnormal impedance or device tilt, and bounded, state-dependent controllers that never exceed clinician-approved parameter envelopes. On the regulatory front, home systems typically require medical-device quality management and software lifecycle compliance, human factors/usability evidence, electrical safety, cybersecurity risk management, and jurisdiction-specific approvals; data handling must satisfy health insurance portability and accountability act/general data protection regulation level protections with explicit teleconsent and audit trails. Pragmatically, payer coverage and procurement pathways, device servicing, and equity (digital access, language, cognitive load) should be evaluated prospectively, ideally via pragmatic, multi-site implementation studies that embed signal-quality audits and adverse-event surveillance alongside clinical endpoints. Translational progress is now expanding these adaptive frameworks beyond the motor disorders. For AD and elderly patients, AI-assisted tDCS systems are under development that will automatically adapt the parameters of stimulation during the stimulation process according to the patients’ cognitive state and reactivity [179]. Earlier phase clinical trials have shown that closed-loop tDCS, using AI developed from EEGs or behavioral markers, can improve memory and attention relative to traditional, fixed parameters. These systems are also investigating coupling with cognitive training, providing possibilities for synergistic neuroplasticity and longer-lasting transfer effects [180,181,182]. In MS, although closed-loop TMS and tDCS paradigms are less developed, the application of adaptive stimulation driven by neurophysiologic biomarkers, such as motor-evoked potentials (MEPs) and fatigue indices, is gaining increasing interest [183]. Different studies recently put AI to work in the online prediction of fatigue or cognitive failure by using this technology in real time to justify personalized, on-demand neuromodulation [184,185]. The greatest challenge remains the heterogeneity and stochasticity of symptomatology onset in MS, highlighting the need for AI methods and tools that can scale to heterogeneous source modalities and maintain up-to-date predictions to adapt to a dynamic disease course [186]. Even though the initial findings are encouraging, there are significant problems in all three diseases. Technical challenges involve the need for reliable, long-term monitoring of neural/physiological state, extreme power and computation constraints in implantable/wearable applications, and the need for artifact reduction during simultaneous sensing and stimulation. Complementary closed-loop architectures for noninvasive stimulation increasingly pair transcranial electrical stimulation (tES)/tDCS with real-time fMRI or EEG to steer dosing toward target network states, an approach that aims to improve durability without adding clinic burden [187]. From a technical standpoint, sustained performance depends on robust stimulation-artifact suppression during concurrent sense-stim operation, stable long-term biomarkers, and low-power computation; recent design studies provide guidance on shielding, blanking, and algorithmic artifact rejection for implantables [178]. Next-generation large-scale clinical adoption will likely be driven by neuromorphic hardware (computer architectures that emulate the structure and operation of the human brain) and federated learning techniques (distributed ML methods that allow model training in multiple centers or devices without sharing patient data) when adaptive systems of greater complexity and modality are involved [188]. Furthermore, neuromorphic chips (event-driven, spiking computation) promise large energy savings for an always-on inference at the edge, while federated learning can train controllers across centers and devices without moving raw patient data, addressing privacy and domain-shift concerns [189,190].

### 4.2. AI-Driven Optimization of Neuromodulation Parameters

A major innovation in the search for precision neuromodulation has been the application of AI-driven algorithms to tuning stimulation parameters, taking what was once considered an “art” based on empiricism to a data science. Manual selection of DBS settings is naturally labor intensive and exhibits large interclinical variability for PD. With the advent of AI, systems now include automated, patient-specific tuning of stimulation protocols to improve efficacy and minimize side effects [191]. The study of Eid et al. [192] describes the evolution and clinical validation of an ML-optimized, lead-free piezoelectric nanoparticle-based DBS (LF-PND-DBS) platform for PD. It is an advanced type of DBS in which piezoelectric nanoparticles, which are biocompatible and lead-free, are used to transfer electric pulses to the exact locations of the brain where they are needed, rather than the conventional metal electrodes. These nanoparticles turn electrical currents on when prompted directly with external events, such as ultrasound. It is the first time wireless and invasive neuromodulation has been achieved without using metallic infiltrates. The goal of this study was to minimize the risk of infections, diminish adverse effects of DBS, and achieve more focused, patient-specific stimulation. Using neural sensing and an ML controller, stimulation parameters were continuously updated to a patient’s real-time electrophysiological signature. LF-PND-DBS was more accurate at identifying PD motor subtypes and resulted in greater shrinking of the Unified Parkinson’s Disease Rating Scale (UPDRS) scores than conventional strategies. Significantly, its leadless design and small device size constitute an important achievement towards safer and more feasible neuromodulation [192]. Boutet and colleagues [193], instead, developed an objective, image-guided methodology to optimize DBS for PD using a unique combination of functional MRI (fMRI) and ML. They proved, in a prospective study of 67 patients, that the clinically optimal DBS settings evoke a characteristic and reproducible brain network activation pattern, which is centered on the motor network. Training a linear discriminant analysis model with these fMRI signatures, the group was able to predict what DBS settings would bring about maximum clinical benefit for individual patients: 88% for optimized cases and more than 75% for stimulation-naïve patients. The results suggest that the network effects of DBS are indeed distributed, involving not only motor but also non-motor brain regions in the optimal response. Although the need for 3 Tesla fMRI (the strength of the magnetic field used) precludes broad application, this method establishes a new rendition of biomarker-informed, personalized neuromodulation. If replicated in substantially larger groups, fMRI-informed, ML-assisted programming might radically change how we treat patients, making DBS safer, more effective, and genuinely personalized [193]. In AD, a significant growth is witnessed in the tDCS parameter optimization methodology design based on AI. EEG, cognitive performance metrics, and patient-reported outcomes can be analyzed with DL models to guide personalized stimulation montages and dosing [194]. In the context of MS, some pilot studies have recently started adopting AI algorithms to choose TMS parameters aimed at individual patterns of demyelination or fatigue. For example, AI-guided analysis of MEPs or functional MRI may enable the adaptation of TMS protocols to provide optimal timing and intensity of intervention, leading to benefits of motor function and fatigue [195]. Despite being early, the translational justification remains high, especially considering the episodic and highly variable nature of MS. In all three cases, AI-guided parameter optimization reduces clinician workload, avoids unwanted side effects, and allows fast reaction to disease evolution or variation in symptoms. Subsequent prospective work shows that “network fingerprints” measured with 3T fMRI under optimal vs. non-optimal settings can train linear discriminant or related classifiers to recommend patient-specific configurations streamlining programming while providing a mechanistic readout of distributed network engagement [193]. In parallel, open-source ecosystems now support parameter search and targeting; Lead-DBS v3.0 enables atlas- and connectome-informed electrode reconstructions and “sweet-spot” mapping, while SimNIBS/ROAST pipelines help personalize tES/TMS fields for noninvasive protocols [194,195]. For sensing-enabled devices, the RC+S data toolbox standardizes high-volume neural time series for downstream ML, accelerating reproducible optimization pipelines [196]. Methodologically, studies span classical supervised learning (e.g., linear discriminant analysis (LDA) for fMRI patterns), deep networks for high-dimensional signals, Bayesian optimization for efficient parameter exploration, and early reinforcement learning prototypes for adaptive control in silico or benchtop settings [172]. Nevertheless, key limitations remain, such as the requirement for big, good-quality datasets to train robust models, external validation in various populations, and the integration with easy-to-use clinical interfaces.

### 4.3. AI for Predictive Modeling and Biomarker Identification

Use of AI-based predictive modeling and biomarker discovery is transformative in the way clinicians stratify patients and choose neuromodulation candidates, as well as in the prediction of treatment response. In the setting of PD, ML-based algorithms built on intraoperative local field potentials, wearable sensor data, and longitudinal clinical parameters can now forecast both short- and long-term outcomes of DBS. They enable better patient characterization and the opportunity to modulate therapy as disease phenotype changes over time [197]. One paper shows how embedded ML controllers may uncover personalized “response fingerprints”, which serve two purposes: (1) identification of PD subtypes and (2) personalization of neuromodulation strategies [198]. AD has also been the beneficiary of the implementation of AI for biomarker discovery and predictive modeling. DL in multimodal data (EEG, MRI, digital cognitive tests) has provided robust biomarkers of disease progression and tDCS/TMS response [199]. For instance, ML-derived features from the EEG were used to predict the likelihood of cognitive enhancement after tDCS and to stratify the sample of patients for interventional trials. These strategies hold the potential to transition neuromodulation from an empirical selection to a biomarker-driven personalization, which would be a significant major shift in a field characterized by clinical heterogeneity [200]. In MS, AI has so far been largely implemented for the development of algorithms for automatic imaging and electrophysiological markers predictive of the efficacy of neuromodulation and the general disease course [201]. Recent investigations have used volumetric MRI, diffusion tensor imaging, and clinical metrics to create ML models to predict which patient may be most responsive to TMS or tDCS and predict risk of relapse or decline in cognition [202,203]. While these strategies are at an earlier stage of translation than in PD or AD, they establish the first steps toward adaptive, biomarker-guided neuromodulation in MS. In every indication, one challenge that research is facing is to become able to have a model that is generalizable and interpretable. The lack of standardization of datasets, small sample sizes, and insufficient multi-center validation are the main obstacles to clinical application. Ethical and regulatory issues, including the interpretability of “black box” AI models (as mentioned before), also need to be resolved as these tools progress toward clinical practice [204]. Building on these foundations, translation now hinges on two intertwined themes: the stability of candidate biomarkers under continuous, real-world sensing and the design of algorithms that can act on those biomarkers in ways that are safe, explainable, and power-efficient. Technically, long-term monitoring is constrained by stimulation/sensing interference, drift, and domain shift across sleep–wake or medication states; recent engineering work details shielding/blanking and algorithmic artifact rejection for concurrent sense-stim systems, while chronic sensing platforms (e.g., Percept™ PC) illustrate how beta-band signals can be captured in vivo with manageable artifact profiles [178,179]. In practice, many predictive pipelines still combine classical supervised models (e.g., LDA/gradient boosting machines (GBMs) with deep nets for high-dimensional signals; community benchmarks for freezing-of-gait (FoG) demonstrate robust CNN/LSTM ensembles and transparent leaderboards that accelerate external validation and regulator-facing endpoint development [132]. For readers wishing to experiment hands-on, open resources include datasets such as Alzheimer’s disease neuroimaging initiative (ADNI)/ADNI4, Parkinson’s progression markers initiative (PPMI), the precisionFDA Freezing-of-Gait Challenge and selected OpenNeuro EEG/TMS sets, and toolchains such as Lead-DBS (DBS imaging pipelines), SimNIBS/ROAST (tES/TMS field modeling), the Summit RC+S toolbox (implantable neural data streaming), and MNE-Python for multimodal signal analysis—enabling transparent benchmarking and reproducible workflows.

### 4.4. Future Perspectives: The Convergence of AI, Neuroimaging, and Smart Biomaterials for Next-Generation Neuromodulation

**Figure 1:**
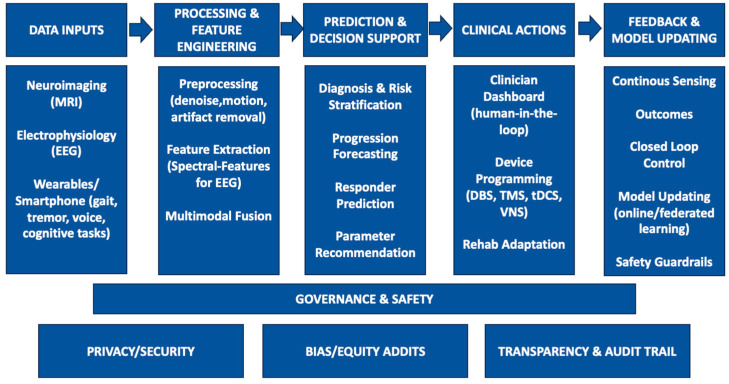
Step-by-step AI pipeline combined with neuromodulation techniques.

The combined power of AI, sophisticated neuroimaging, and intelligent biomaterials is ushering in the era of intelligent neuromodulation in various forms of neurodegenerative conditions. As a unifying blueprint, future systems will integrate (i) robust biomarkers (EEG, kinematics), (ii) interpretable controllers that expose rationale to clinicians (e.g., feature-attribution or rule-based overlays) to bolster trust, and (iii) secure analytics that learn continuously from real-world deployment [171]. Figure 1 presents the step-by-step AI pipeline, data inputs, processing, prediction/decision support, and feedback, clarifying how it connects upstream data to clinical outputs and integrates with neuromodulation across neurodegenerative diseases. In PD, AD, and MS, combining real-time neural/behavioral feedback, imaging-based network structures, and flexible, miniaturized stimulators will yield autonomous, adaptive systems that could learn and change with each patient [205]. The continued progress of several technologies, including neuromorphic computing, federated learning, and multi-modal sensor integration, will enable evermore powerful AI algorithms on power- and resource-constrained platforms, adding secure data management, strong privacy protections, and effortless scalability, even in more remote locations [206]. Concretely, neuromorphic processors can cut energy per inference by orders of magnitude via event-driven spiking networks, critical for always-on classifiers in implants and wearables, while federated learning allows sites/devices to co-train models without sharing raw data, mitigating privacy risk and site bias [190,191,207]. Most importantly, next-generation intelligent biomaterials will serve as durable and safe implants that are biocompatible and responsive to the normal physiologic stimuli for prolonged therapeutic life spans [208,209]. Other future priorities include artifact-robust sensing, standardized reporting of control policies, prospective multi-site validation with meaningful horizons (≥6–12 months), and cost-effectiveness analyses embedded in deployment studies. If the potential of such transformative technologies is to be realized, the field will need to emphasize tight clinical validation, transparent and patient-centric design, and sustained multidisciplinary collaboration between clinicians, engineers, data scientists, and regulators. In the end, intelligent neuromodulation could serve to not only increase efficacy but also increase access and quality of life for everyone afflicted with a neurodegenerative disease, reshaping standards of care in the next decade.

## 5. Translational and Clinical Implications of AI-Enhanced Neuromodulation in AD, PD, and MS

In the earlier paragraphs we have seen that AI combined with neuromodulation has the potential to upend the care of PD, AD, and MS in the clinic, ushering in a more explicitly personalized, responsive, patient-centric treatment paradigm. Beyond device engineering or algorithmic performance, the central translational impact is a reframing of neurological care, not merely a new option for a subset of patients but a systematic shift toward learning and data-informed practice. Among the most obvious clinical implications is the shift away from treatment protocols to more individualized care pathways. Neuromodulation systems with AI are able to incorporate a variety of patient data streams, imaging, genetics, digital biomarkers, and real-time physiological signals to assemble richer patient profiles and modify interventions in real time. Practically, this implies that patients with PD can receive software updates to change DBS settings as their symptoms and/or responses to medication change over time [210], people with AD could be offered state-dependent tDCS or TMS protocols that change based on changes in their cognition and/or behavior [211,212], and patients with MS may have better management of their fatigue, cognitive dysfunction, and/or motor decline through programs that will automatically adjust to their state according to recent experience [213]. Extending across inpatient and outpatient settings, AI-enabled remote monitoring and tele-neuromodulation can avoid frequent hospitalization, support earlier intervention at early stages of deterioration, and engage patients more actively during their disease [214]. In parallel, clinical workflows are poised for reconfiguration, with AI-driven documentation, decision support, and predictive analytics automating standard functions and allowing the clinician to devote attention to higher-value, complex care [215]. To guide this transition coherently, we articulate two linked themes below.

### 5.1. Patient-Level Personalization and Connected Care

From a guideline development perspective, the insights generated by large-scale, real-world deployment of AI-driven neuromodulation will be critical. Real-time and longitudinal data streams can be aggregated to iteratively refine guidelines used to identify and adopt best practices and to develop bespoke evidence-based adaptive protocols, which can be implemented across care in PD, AD, and MS. To achieve this, data standards will need to be unified, multi-site validation will have to be rigorous, and infrastructure will need to be robust and interoperable to ensure secure data use [216]. Moreover, patient-facing implementations should include clear escalation rules, monitoring thresholds, and clinician-in-the-loop oversight so that adaptive adjustments remain transparent and clinically actionable across settings. Building on this framework, connected pathways should couple shared decision-making dashboards (integrating symptoms, digital biomarkers, and device telemetry) with caregiver inputs, enabling clinicians to reconcile objective signals with perceived benefit and burden [217]. To keep adaptations safe and interpretable, sites can predefine decision boundaries (e.g., symptom- and biomarker-triggered titration bands), hard stop rules for adverse events, and time-bounded review cycles for every algorithmic change, each documented with version control and audit trails. Finally, to ensure equitable uptake, programs should incorporate human factors and accessibility testing (language, vision, and motor accommodations), offer digital literacy support, and define caregiver delegation pathways for device use and troubleshooting. Pragmatic, hybrid effectiveness implementation studies can evaluate these elements together, linking patient-centered outcomes (falls, function, participation), health-service key performance indicators (unplanned visits, time-to-titration), and safety indicators, so that living guidance evolves in tandem with real-world deployment rather than lagging behind it [218].

### 5.2. Standards, Ethics, and Health System Adoption

Furthermore, the social and ethical ramifications are not to be understated. AI-enabled personalization heightens the importance of privacy preservation, algorithmic transparency, and consent models tailored to cognitively vulnerable populations. There is a need to advocate for interpretable and trustworthy uses of AI and for transparent descriptions of how data are used and decisions made to patients and their families [219]. Proactive bias auditing and representative training data are required to avoid inequities in access or outcomes between ethnic groups [220]. Economically, combining AI and neuromodulation represents a challenge and an opportunity. Although advanced adoption and training costs are high, credible long-term value is plausible (via fewer hospitalizations, better resource use, and improved quality of life). Accordingly, health systems will need to develop reimbursement and delivery models that recognize the value of remote monitoring, adaptive treatment, and continuous improvement with data [221,222,223]. In the end, successful clinical application of AI-guided neuromodulation will rely on ongoing collaboration among clinicians, scientists, engineers, ethicists, and policymakers. Sustained workforce training, public education, and transparent governance should be prioritized to ensure equitable access to these advancements [224]. Taken together, these steps create a realistic pathway from innovation to impact, opening the door to AI-driven neuromodulation that enhances autonomy, independence, and daily functioning for people living with neurodegenerative disease.

## 6. Discussion: Critical Reflections on the Path to Personalized Neurorehabilitation in Neurodegenerative Disorders

The evidence reviewed in this narrative review supports a convergent thesis; coupling AI with neuromodulation can shift neurorehabilitation for AD, PD, and MS from static, protocol-driven interventions to adaptive, data-informed, and patient-specific care. This is not merely an incremental upgrade to existing tools, it is a reframing of how we sense disease states, decide on stimulation parameters, and evaluate outcomes. In particular, continuous physiological and behavioral sensing, algorithm-enabled inference, and clinician-supervised control close a loop that promises to align therapy with each patient’s evolving neural and functional profile. The ultimate goal of precision neurorehabilitation that is effective, scalable, and equitable comes into clearer view, but the path to it requires careful attention to mechanistic biomarkers, algorithmic safety, device engineering, workflow integration, ethics, and regulatory science. A balanced reading of the literature suggests uneven maturity across disorders and modalities. In PD, aDBS anchored to subthalamic beta-band dynamics has progressed from short-term demonstrations to chronic, ambulatory use with signals of superior motor control and improved energy efficiency relative to conventional DBS [169,175,176,177]. In contrast, adaptive noninvasive paradigms in AD and MS, such as EEG- or behavior-informed tDCS/TMS, are earlier in development, with preliminary trials showing feasibility and short-term cognitive or fatigue benefits compared to fixed dosing [180,181,182,186]. Across indications, connected-care models that pair remote monitoring with clinician-in-the-loop titration appear particularly tractable, offering a near-term route to reduce hospitalizations and accelerate adjustments while maintaining oversight [145,161,219]. Importantly, these translational gains are not purely technical; they hinge on aligning measurement, modeling, and intervention around outcomes that matter to patients (mobility, cognition, and participation) rather than surrogate signals alone.

Mechanistic clarity is pivotal for durable personalization. Candidate biomarkers, subthalamic nucleus beta power in PD, perturbational TMS-EEG indices in AD-relevant networks, motor-evoked potentials, and fatigue signatures in MS anchor closed-loop policies to neurophysiology with plausible causal relevance [175,180,184,186]. Yet, biomarkers must be stable enough for control, sensitive to clinically meaningful change, and robust to context (sleep–wake cycles, medication state, and daily activity). This demands longitudinal validation under real-world conditions with attention to domain shift and signal drift. It also argues for multi-scale sensing-neural (EEG), kinematic, and ecological (speech, activity), that can triangulate state while mitigating the brittleness of any single channel. In this regard, the field benefits from open toolchains and resources that promote reproducible biomarker discovery and external validation, including Lead-DBS for DBS reconstructions and network mapping, electric-field modeling for noninvasive stimulation, the RC+S data toolbox for sensing-enabled devices, and community benchmarks for freezing-of-gait detection [132,195,196,197].

At the algorithmic level, safety and interpretability are design constraints, not afterthoughts. Supervised and deep learning models can map high-dimensional signals to state estimates or outcome forecasts, but clinical adoption depends on calibrated probabilities, uncertainty quantification, and human-interpretable rationales for parameter changes [166]. For control, reinforcement-learning or Bayesian policies are attractive because they optimize longer-horizon utility under uncertainty; however, they must operate within hard safety envelopes (dose limits, lockouts, adverse-event “hard stops”) and display simple, auditable rules (for example, dual-threshold hysteresis on beta power) that clinicians can verify and override [172,175,178]. The versioning of models and policies, with audit trails linking input distributions to decisions, will be necessary for both trust and regulatory review. Finally, algorithmic fairness cannot be appended later; representative training data, bias audits by subgroup, and pre-specified performance parity thresholds should be baked into development and reporting [220]. Device and computation constraints shape what is feasible at the bedside. Implantables and wearables must acquire clean signals during stimulation, suppress artifacts, and run inference at low power for months to years. Practical solutions include hardware blanking and shielding, adaptive filtering synchronized to stimulation pulses, and compact models optimized for edge inference [178]. Neuromorphic processors and event-driven architectures promise substantial energy savings for always-on classifiers, while federated learning enables cross-site model updates without centralizing raw patient data, mitigating privacy risk and site bias [189,190,191,207]. Even with these advances, rigorous engineering verification, latency budgets, failure-mode effects analyses, and cybersecurity threat modeling remain essential for safety-critical systems.

Translation will ultimately be determined by how well these technologies are embedded in day-to-day clinical workflows. Clinician-in-the-loop designs should specify escalation pathways, monitoring thresholds, and titration “bands” in language that maps to routine documentation and order sets. Shared dashboards that integrate symptoms, digital biomarkers, and device telemetry, augmented by caregiver inputs, can reconcile objective signals with perceived benefit and burden, making adjustments defensible and transparent [217]. Implementation also requires role delineation (neurology, rehabilitation, nursing, engineering), training curricula, and competency tracking so that device programming and data interpretation are not limited to a small cadre of experts. In parallel, integration with electronic health records, problem lists, medication timelines, and adverse-event logs will reduce swivel-chair burden and enable closed-loop learning at the health system level.

Ethical and equity considerations are inseparable from clinical effectiveness. Many patients with neurodegenerative disease have cognitive vulnerability, which challenges conventional consent and disclosure practices. Consent processes should be staged and reinforced, combining plain-language explanations with visualizations of how data flow and decisions are made, and should recognize caregiver delegation where appropriate [219]. Privacy-preserving architectures (local preprocessing where feasible, encrypted telemetry, and federated learning) and explicit data minimization can reduce risk [207]. Equity demands human factors testing across language, vision, and motor abilities; accessible interfaces; and support for digital literacy. Programs must also budget for device replacement, technical support, and loaned equipment to avoid differential dropout by socioeconomic status. Without such guardrails, algorithmic personalization risks amplifying, rather than narrowing, existing disparities; fairness frameworks should therefore be applied prospectively in health–AI studies [155,171,220]. The regulatory and reimbursement landscape is evolving but not yet aligned with adaptive therapeutics. Regulators increasingly recognize “software as a medical device” and are piloting pathways for learning systems, but change-control protocols, real-world performance monitoring, and cybersecurity requirements remain moving targets [168,218,222]. For neuromodulation, clear pre-specification of control policies, safety constraints, and update triggers will facilitate review. Post-market commitments, registries, adverse-event surveillance, and periodic recalibration audits should be expected for adaptive systems, much as with other implantable technologies. On the financing side, fee-for-service models undervalue remote monitoring and iterative titration, whereas bundled or value-based arrangements can reward reductions in hospitalizations and improved function; early organizational surveys suggest that readiness varies widely across health systems, reinforcing the need for economic endpoints in trials [221,223]. Given these system-level dependencies, implementation science is not optional. Hybrid effectiveness–implementation trials can evaluate clinical outcomes and deployment metrics in tandem, covering reach, adoption, fidelity, and cost, accelerating the cycle from pilot to scale. Multicenter consortia with shared data standards will be required to test generalizability across devices, sites, and populations; MS provides a model as stakeholders coalesce around an AI-driven personalized care agenda with harmonized pipelines and governance [216]. Core outcome sets, patient-reported function, participation, and quality-of-life, alongside clinical scales and device metrics, can reduce heterogeneity and support meta-analysis. Throughout, reporting standards should require explicit descriptions of control policies, artifact-handling procedures, and data-drift monitoring to make replication feasible [218].

A critical appraisal of the current evidence identifies notable strengths and recurrent limitations. Strengths include maturing chronic aDBS in PD with mechanistically grounded biomarkers, the emerging feasibility of adaptive tDCS/TMS in AD and MS, and a growing ecosystem of open benchmarks and toolchains that support reproducibility [132,169,175,176,177,180,181,182,195,196,197]. The limitations are familiar: small samples, single-center designs, short follow-up, heterogeneous protocols, and inconsistent external validation [132,134,157,166,218]. Many studies rely on surrogate endpoints without parallel measures of real-world participation or caregiver burden, and adverse-event surveillance is often underpowered. Algorithmic components are too rarely preregistered, and model updates across time are seldom documented with performance-over-shift analyses. These gaps do not negate the observed signals of benefit, but they do caution against overgeneralization and emphasize the need for rigorous, transparent methods. Against this backdrop, a pragmatic agenda emerges. Firstly, prospective, multicenter trials with ≥6–12-month horizons should report patient-centered outcomes, stimulation-artifact handling, energy and use metrics, and blinded adjudication of adverse events. Secondly, head-to-head comparisons (adaptive vs. fixed dosing; clinician-programmed vs. AI-assisted) are needed to quantify incremental value. Thirdly, external validation across scanners, sensors, and demographics should be mandatory before clinical deployment, with prespecified subgroup analyses to assess fairness. Fourthly, methodological work should prioritize interpretable controllers with formal safety guarantees (e.g., constrained reinforcement learning), neuromorphic edge computing for low-power inference, and federated-learning pipelines with robust privacy and auditability [172,189,190,191,207,218]. Fifthly, human factors research must optimize home and tele-supervised use, montage verification, impedance checks, skin-integrity prompts, and clear lockout rules, so that safety is maintained outside the clinic [90,94]. Finally, economic evaluations embedded in implementation trials can inform reimbursement models that sustain programs beyond grants [221].

Clinical applications should be sequenced by readiness and risk. Short-term, “low-regret” uses include AI-assisted decision support for DBS programming, clinician-supervised remote monitoring with predefined escalation rules, and tele-supervised home tDCS within strict parameter envelopes, each leveraging existing infrastructure while accumulating real-world evidence [90,93,94,145,192]. Intermediate applications encompass semi-adaptive noninvasive stimulation guided by EEG or behavioral markers for cognitive support in AD or fatigue in MS, implemented via registries and learn-as-you-go protocols under close oversight [181,214]. Longer-term ambitions of fully adaptive noninvasive systems and next-generation implants with on-device learning will require durable biomaterials, ultra-low-power inference, and mature regulatory paradigms, but they are plausible endpoints of the current trajectory [189,190,191,207,211,212]. Throughout, disease-specific nuances matter; PD offers well-defined oscillatory targets and established implantable platforms, AD demands sensitive cognitive biomarkers and rigorous consent frameworks, and MS emphasizes episodic dynamics, heterogeneity, and accessibility for patients with variable motor and cognitive loads. The field’s forward momentum will depend on collaborative standard-setting; joint statements on reporting, safety envelopes, fairness metrics; and shared infrastructure that lowers the barrier to multicenter studies. In parallel, a cultural shift toward preregistration, code and model sharing, and independent replication will help guard against hype cycles and ensure that the most promising advances translate into reliable clinical gains.

## 7. Conclusions

This narrative review synthesizes converging evidence that the integration of artificial intelligence with neuromodulation, across DBS, TMS, tDCS, and VNS, can move care for AD, PD, and MS from static, protocol-driven approaches toward adaptive, data-informed, and patient-specific interventions. Advances in sensing (neural, kinematic, and behavioral), multimodal analytics, and interpretable control policies are enabling closed-loop systems that titrate stimulation to symptom and network state in real time. Early signals of benefit are most mature for adaptive DBS in PD, with emerging applications in AD and MS; in parallel, connected-care models that pair remote monitoring with clinician-in-the-loop titration suggest a practical route to scale, access, and sustained engagement. The clinical implications are immediate. Implementation will hinge on standardized data pipelines, explicit safety guardrails, and transparent oversight so that algorithmic decisions remain auditable and clinically actionable. Embedding AI-guided neuromodulation within multidisciplinary pathways, spanning inpatient to home settings, can streamline documentation, decision support, and follow-up while preserving shared decision-making and equity. Governance frameworks that prioritize privacy, model interpretability, and inclusive consent are essential, particularly for cognitively vulnerable populations, and must be coupled with workforce training and patient/caregiver education. A focused research agenda should now prioritize (i) prospective, multi-site trials with meaningful horizons (≥6–12 months) that report patient-centered outcomes, adverse-event surveillance, and energy/use metrics; (ii) standardized reporting of control policies and stimulation-artifact handling to enable replication; (iii) robust external validation of biomarkers under real-world sensing, with attention to domain shift; and (iv) comparative effectiveness and cost–utility analyses to inform reimbursement and equitable access. Methodologically, promising directions include reinforcement-learning and Bayesian controllers with safety constraints, neuromorphic edge computation for low-power inference, federated learning for privacy-preserving model updates, and smart biomaterials that extend device longevity and biocompatibility. On the practice side, priorities include interoperable electronic health record integration, registries for post-market surveillance, clear escalation/lockout rules for home-based use, and co-design with patients and caregivers to ensure usability across language, vision, and motor abilities. If pursued with rigorous validation, transparent design, and sustained multidisciplinary collaboration, AI-enhanced neuromodulation can deliver not only incremental efficacy but also broader access and better day-to-day function, bringing precision neurorehabilitation within reach for people living with neurodegenerative disease.

## Figures and Tables

**Table 1:** Key clinical and practical aspects of major neuromodulation techniques in neurodegenerative disorders.

Neuromodulation Technique	Imaging/Biomarker Guidance	Patient Suitability/Contraindications	Combination with Other Therapies	Unique Adverse Effects in Neurodegenerative Disorders (NDs)	Remote Monitoring/Telemedicine	Typical Session Duration/Frequency
Deep Brain Stimulation (DBS)	This technique utilizes MRI and microelectrode guidance as well as local field potentials for adaptive stimulation to optimize targeting and outcomes [109].	DBS is generally not suitable for individuals with significant cognitive impairment, severe psychiatric comorbidities, or pronounced frailty. Careful patient selection is especially important in elderly populations [29,39].	DBS has shown evidence of synergy when used alongside medication and physiotherapy, and it is currently being investigated in combination with cognitive rehabilitation protocols for AD [29,33].	Patients may experience surgical risks such as infection and hemorrhage, and there is also the potential for neuropsychiatric effects. If the electrode targeting is not optimal, there is a risk of worsened cognition [29,40,41].	There is a growing possibility for remote adjustment and monitoring of DBS devices, allowing for improved follow-up and patient management [39].	The procedure requires a multi-hour surgical implantation, followed by regular programming sessions and long-term device management [29,32].
Transcranial Magnetic Stimulation (TMS)	TMS often relies on MRI navigation, and in some studies, EEG or cognitive biomarkers are employed to guide stimulation protocols for enhanced efficacy [56].	TMS is contraindicated in individuals with epilepsy, metal implants, or unstable medical conditions, and should be used with caution in advanced dementia [42,56].	It Is frequently combined with cognitive training in AD and with physical rehabilitation in PD and MS. There is also potential synergy with pharmacological treatments [43,44,51,52].	The most common adverse effects include transient headache and discomfort. There is a rare risk of seizure, and agitation may occur in individuals with severe dementia [42,56].	Currently, TMS is primarily clinic-based. Home or remote use is limited but represents an ongoing area of research [56].	Typical treatment consists of sessions lasting twenty to forty minutes, administered daily or several times per week for a series of weeks. Maintenance protocols may be used for chronic NDs [42].
Transcranial Direct Current Stimulation (tDCS)	Some clinical trials utilize EEG or cognitive biomarkers, and there is emerging use of artificial intelligence for the optimization of stimulation parameters [76].	tDCS is generally well tolerated, but caution is advised in patients with epilepsy or unstable cardiac conditions. Data on its use in severe dementia are limited [67,81].	It is commonly combined with cognitive or physical training and there is strong evidence supporting additional benefits in AD, mild cognitive impairment, and PD. The technique is also feasible for use in the home setting [73,74,81].	Adverse effects are usually mild, including skin irritation or tingling. In rare cases, confusion may develop in elderly patients with cognitive impairment [67].	There is high potential for mobile or home-based tDCS systems, with development of apps and cloud data integration for supervision and monitoring [67,81].	Sessions typically last twenty to thirty minutes, are performed daily or several times per week, and are often continued for several weeks or months [67,70].
Vagus Nerve Stimulation (VNS)	Cardiac monitoring is used to trigger stimulation in some cases, and there is exploratory research on using EEG and neurochemical markers to guide therapy [95,96].	Surgical VNS should be avoided in patients with severe cardiopulmonary disease or coagulopathy. Non-invasive VNS is currently under evaluation for broader patient populations [104,105].	Early evidence suggests synergy with task-specific rehabilitation with pharmacotherapy for PD. There is also some investigation into combining VNS with cognitive training [20,93,100,106,108].	Adverse effects may include hoarseness, cough, and vocal changes in patients with implanted devices, as well as surgical risks. Rarely, patients may experience bradycardia or dyspnea [104,105].	Non-invasive VNS devices are increasingly compatible with remote use, and remote patient monitoring is under active investigation [104].	Implanted VNS requires regular surgical check-ups, whereas non-invasive VNS may involve multiple daily sessions that can be performed at home [105].

Legend: neurodegenerative disorders (NDs), deep brain stimulation (DBS), magnetic resonance imaging (MRI), Alzheimer’s disease (AD), transcranial magnetic stimulation (TMS), electroencephalography (EEG), Parkinson’s disease (PD), multiple sclerosis (MS), transcranial direct current stimulation (tDCS), vagus nerve stimulation (VNS).
